# The effect of *Lavandula Coronopifolia* essential oil on the biophysical properties of desensitization and deactivation gating currents in ionotropic receptors

**DOI:** 10.1038/s41598-023-35698-0

**Published:** 2023-05-24

**Authors:** Mohammad Qneibi, Nidal Jaradat, Nawaf Al-Maharik, Mohammed Hawash, Linda Issa, Shorooq Suboh, Leen Yahya, Adan Abu Khait, Amjaad Warasneh, Sosana Bdir

**Affiliations:** 1 Department of Biomedical Sciences, Faculty of Medicine and Health Sciences, An-Najah National University, Nablus, Palestine; 2 Department of Pharmacy, Faculty of Medicine and Health Sciences, An-Najah National University, Nablus, Palestine; 3 Department of Chemistry, Faculty of Sciences, An-Najah National University, Nablus, Palestine

**Keywords:** Membrane biophysics, Diseases, Medical research, Neurology, Chemistry

## Abstract

The rising incidence of cancer and the lack of effective therapeutic interventions for many neurological illnesses like Alzheimer's and epilepsy has prompted us to investigate the composition and effects of the *Lavandula coronopifolia* oil from Palestine on cancer cells and AMPA receptor subunits in the brain due to the vast range of beneficial properties of *Lavandula coronopifolia* essential oil (EO). GC/MS was used to analyze *L. coronopifolia's* EO chemistry. EO's cytotoxicity and biophysical effects on AMPA receptors were investigated using MTS and electrophysiological techniques. The GC–MS results revealed that *L. coronopifolia* EO has a high content of eucalyptol (77.23%), *β*-pinene (6.93%), and α-pinene (4.95%). The EO showed more significant antiproliferative selectivity activities against HepG2 cancer cell lines than HEK293T cell lines with IC_50_ values of 58.51 and 133.22 µg/mL, respectively. The EO of *L. coronopifolia* affected AMPA receptor kinetics (desensitization and deactivation) and preferred homomeric GluA1 and heteromeric GluA1/A2 receptors. These findings indicate the potential therapeutic use of *L. coronopifolia* EO in the selective treatment of HepG2 cancer cell lines and neurodegenerative diseases.

## Introduction

Botanical therapies and herbal supplements have expanded dramatically over the past few years. For many years now, essential oils (EOs) have been extracted from aromatic plants to produce an extract containing various volatile compounds^1^, Terpene, and phenolic contents^2^.

*Lavandula* (often known as lavender) is a genus that has 45 species that are primarily found in tropical and subtropical climates worldwide. For thousands of years, herbals from this genus have been utilized in alternative medicine to cure migraines, headaches, and pain, guided by their anti-flatulence, antirheumatic, antidiuretic, and antiepileptic properties, among many other things. They became well-known for their medicinal, cosmetics, and culinary benefits^3,4^.

*Lavandula coronopifolia* Poir is a perennial hairy small shrub-like herbaceous plant with an acrid aromatic odor. It grows in desert plains and rocky environments, mainly in tropical and subtropical regions. The plant leaves have two or three pinnatisect with oblong-linear and acute lobes^5^.

Many hydroxyl flavones, such as luteolin, isoscutellarien, and hypolaetin, were isolated and identified from dried aerial parts of *L. coronopifolia* by El-Garf et al. in 1999. This was the first time that *L. coronopifolia* was given serious consideration^6^. Many significant biological activities were found in *L. coronopifolia,* such as hepatoprotective^7^, antimicrobial^8^, antioxidative^9^, and antidiabetic^10^ properties.

An evaluation in 2018 by the World Health Organization (WHO) showed that about 18 million tumor cases and 9.5 million tumor deaths occurred globally. Despite progress in cytotoxicity research, complex challenges still hinder finding a cure^11^. The WHO has reported that around 35% of cancer mortality is associated with human nutrition. Hundreds of substances derived from plants have a role in prophylaxis, from the mutation of normal cells to malignant ones.

Lavender oil has been shown to increase the inhibitory tone of the nervous system, as well as have a neuroprotective impact against cerebral ischemia, where excitotoxicity is caused by oxygen deprivation in the brain. It is also used to treat Alzheimer's disease (AD), which is marked by a decline in the activation of the α-amino-3-hydroxymethyl-4-isoxazolyl-propionic acid receptors (AMPARs) and synapses loss^12–15^. More so, in the case of cerebral ischemia, AMPARs were implicated in the permeability of the blood–brain barrier^16^. *Lavandula officinalis*, another kind of lavender, contains antiepileptic properties that inhibit glutamate release in the central nervous system^17^.

AMPARs are tetrameric receptor assemblies with four unique subunits (GluA1-GluA4) expressed in homomeric or heteromeric configurations that provide functional variety and determine AMPARs trafficking^18^. Because heteromerization is a crucial mechanism for controlling AMPA receptor functions and dynamics, most AMPAR subunits assemble as heteromers rather than homomers^19^. Each AMPA receptor subunit comprises an extracellular amino-terminal domain, three transmembrane domains, and an intracellular carboxyl-terminal domain. The extracellular domain contains the binding site for glutamate and other ligands, while the transmembrane domains form the ion channel through which ions pass in response to ligand binding. The carboxyl-terminal domain is responsible for various protein–protein interactions and post-transcriptional modifications that regulate the activity and trafficking of the receptor^20^.

The brain's AMPARs play an essential role in rapid excitatory transmission, where any alteration in their number or function leads to long-term changes in synaptic plasticity. The excitatory postsynaptic current time course shows that fast clearance of neurotransmitters from the synaptic cleft is associated with rapid deactivation of AMPARs at numerous central synapses. Additionally, a function in regulating the neurotransmission, particularly during periods of high-frequency synaptic activity or delays glutamate clearance, is played by AMPARs desensitization in the presence of glutamate bound to AMPA receptors. AMPARs need to recover from desensitization before they can be reactivated. Consequently, desensitization and recovery of AMPARs influence the neuronal firing field's amplitude, duration, and frequency^21^.

The pathogenesis of numerous neurological and psychiatric disorders, including but not limited to epilepsy, stroke, Parkinson's disease, Alzheimer's disease, and depression, has been linked to disrupted AMPAR function. Excessive activation of AMPARs can cause excitotoxicity and neuronal damage, leading to the development of these disorders. In contrast, impaired AMPAR function may contribute to the development of depression. Hence, investigating AMPAR regulation mechanisms and identifying novel agents that modulate their activity are crucial for developing effective therapeutics for these disorders^22,23^.

Avoiding AMPAR overactivation is critical for maintaining healthy neuronal function. This may be accomplished by using chemical or natural inhibitors of AMPAR kinetics. This research examines the chemical composition of the essential oil produced from *Lavandula coronopifolia* leaves and its possible cytotoxic effects. Additionally, we want to understand how essential oils affect AMPARs and how they affect their kinetics. Our results will illuminate the therapeutic potential of *L. coronopifolia* essential oil as an AMPAR activity modulator.

## Experimental methods

### Plant materials and essential oil extraction

The leaves of *Lavandula coronopifolia* were obtained from the Jenin governorate of Palestine; the *Lavandula coronopifolia* is not protected or regulated in Palestine because it is wild, and there is no legislation prohibiting its research. The plant leaves of *Lavandula coronopifolia* were collected by WHO standards for evaluating herbal medicines and legislation. All methods followed applicable institutional, national, and international guidelines and legislation. Dr. Nidal Jaradat, a pharmacognosist at An-Najah National University, with a voucher specimen code of pharm-PCT-1367, performed the plant identification and deposition in the Pharmacognosy Laboratory. After the leaves were washed entirely, *L. coronopifolia* plant EO was extracted by hydro-distillation^24^; 1 L of distilled water suspended 0.1 kg of fresh aerial parts. The EO was extracted utilizing hydro-distillation with Clevenger apparatus using air pressure at 100 °C for 150 min. Calcium carbonate was utilized in the chemical purification procedure to preserve the *L. coronopifolia* EO, held at 2–8 °C until further usage, and the yield was 2.15% of the total weight.

### Gas chromatography/mass spectrometry (GC–MS)

The EO components of the *L. coronopifolia* plant were analyzed using a Perkin Elmer Clarus 500 gas chromatograph. This machine was connected to Perkin Elmer Clarus 560 mass spectrometer. The Perkin Elmer Elite-5 fused silica capillary column (film thickness 0.25 µm, 30 m × 0.25 mm) was utilized for separation. At a 4 °C/min rate, the column temperature rose from 50 °C for 5 min to 280 °C. All chromatographic runs were performed with helium flowing at a 1 mL/min rate. 0.2 µl of purified *L. coronopifolia* EO was inserted in split mode with a splitting ratio of 1:50 and at 250 °C. Mass spectra matched the sample components with those in the library or hygienic standards. GC retention durations and indices corroborated the matches^25^.

### Cytotoxicity assay

Cervical cancer (HeLa), hepatocellular carcinoma (Hep3B & HepG2), breast cancer (MCF-7), and human embryonic kidney (HEK293T) cells (ATCC, Rockville, MD, USA) were grown in RPMI-1640 media and supplemented with 1% Streptomycin/Penicillin, 1% l-glutamine, and 10% fetal bovine serum. Before seeding 2.5 × 10^4^ cells per well in 96-well plates, the cells were cultured at 37 °C in a 5% CO_2_ environment. All the measured EO concentrations (500, 300, 100, 50, and 10 µg/mL) were examined 24 h after 48 h of culture. The manufacturer's guidelines (Promega Corporation, Madison, WI) were followed to assess the viability of the cells examined using the CellTilter 96®Aqueous One Solution Cell Proliferation (MTS) Assay. The procedure was completed by adding 20 μL of MTS solution per 100 μL of media and incubating the medium at 37 °C for two h. At 490 nm, the absorbance was measured^26,27^.

### Electrophysiological recordings

All AMPAR-subunit constructions in this investigation were made using the flip isoform. The templates for GluA1-3 (Q-form/flip) were initially provided by S.F. Heinemann (Salk Institute, La Jolla, CA). HEK293T cells (secured from Sigma, Germany) were prepared for transfection, growing in Dulbecco Modified Eagle Medium (DMEM) (Sigma, USA) containing 10% FBS (fetal bovine serum), 0.1 mg/ml streptomycin, and 1 mM sodium pyruvate (Biological Industries; Beit-Haemek, Israel), supplementing the medium with incubation at 37 °C and 5% CO2. A downstream internal ribosome entry site was used to introduce wild-type AMPARs DNA into the pRK5 plasmid designed to create an enhanced green fluorescent protein (EGFP; Clontech, Palo Alto, CA) with a 1:9 cotransfection ratio (pEGFP-C1: GluA subunit), the protein encoded in GFP vector with its regulatory elements for efficient expression in HEK293T cells. We performed transient transfections of HEK293T cells with the plasmid DNA using jetPRIME (Polyplus: New York, NY), as previously explained in our work^28–31^. Cells rested for 36 h before electrophysiology recordings or stereomicroscope imaging by replating them on coverslips coated with Laminin (1 mg/mL; Sigma, Germany), cells exhibiting the most fluorescence were selected. Whole-cell (patch-clamp) current recordings were collected using Integrated Patch Clamp Amplifiers with Data Acquisition System (IPA, Sutter Instruments, Novato, CA) and a rapid solution exchange system that was achieved by using a piezoelectric translator (Automate Scientific, Berkeley, CA) controlling a two-barrel theta glass pipette. One barrel contained the external (wash solution), and the other contained the *L. coronopifolia* EO solution with added glutamate (10 mM). The extracellular solution contained 2.8 mM KCl, 150 mM NaCl, 2 mM CaCl_2_, 0.5 mM MgCl_2_, and 10 mM HEPES, all of which had been adjusted to pH 7.4 using NaOH. Borosilicate glass was used to fabricate patch electrodes; the pipette solution was filled with 110 mM CsF, 30 mM CsCl, 4 mM NaCl, 0.5 mM CaCl_2_, 10 mM EGTA, and 10 mM HEPES. It was adjusted to pH 7.2 using CsOH, and the electrode resistance was 2–4 MΩ. The timing and solution exchange rate was calculated from junction potentials at the open tip of the patch pipette after recordings and were generally between 200 and 300 us (10–90% rise time). Two exponentials fitting the current decline from 90 to 95 percent of the peak to the baseline current were used to calculate the time constants for deactivation (τ_w_ deact) and desensitization (τ_w_ des). The weighted tau (τ_w_) was derived as τ_w_ = (τf x af) + (τs x as), where af and as are the amplitudes of the fast (τf) and slow (τs) exponential components, respectively. We measured the currents of deactivation and desensitization using 10 mM of agonist (glutamate) for 1 ms and 500 ms, respectively. At − 60 mV potential, pH 7.4, and room temperature (20–23 °C), electrical current was recorded at a high sampling rate by setting the frequency to 10 kHz, and high-frequency noise was filtered through a low pass filter setting to 2 kHz, digitized by SutterPatch Software v. 1.1.1 (Sutter Instruments). All tests were performed in different cells collected from at least 7–9 independent transfections (separated in time). We employed Igor Pro7 (Wave Metrics, Inc) for our data analysis. The supplementary material includes recorded whole-cell recordings and complete data analysis (Table S1).

### Statistical analysis

Statistical differences between the groups and the wild type were examined by one-way analysis of variance (ANOVA), and the significance was set as * *p* < 0.05; ** *p* < 0.01; ns, not significant, with values of *** *p* < 0.05 being considered to indicate statistical significance. The number of tested HEK293T cells by the *Lavandula Coronopifolia* oil (n = 8) is presented as mean ± SEM. Concentration–response relationships were fitted as composite curves using GraphPad Prism version 6.01 (GraphPad Software) to the Hill equation. All results were representative of at least three independent experiments.

## Results

### *Lavandula coronopifolia* essential oil components

**Figure 1:**
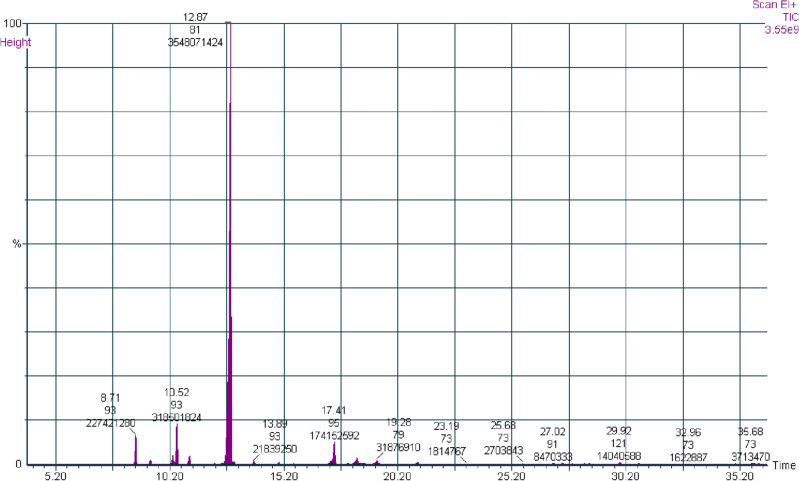
GC–MS chromotogram of *Lavandula coronopifolia* essential oil.

The GC–MS analysis of *L. coronopifolia* EO identified sixteen components; Table 1 represents 100 percent of the chromatographic area. Figure 1 shows the separation of the most abundant components of eucalyptol (1,8-cineole), β-pinene, α-pinene, and camphor based on their retention times. The x-axis of the chromatogram would represent time, while the y-axis would represent the intensity of the signals detected by the mass spectrometer. Each peak in the chromatogram would correspond to a specific compound in the sample. The height and area of the peak would be proportional to the concentration of the compound in the sample. In this case, the most abundant compound, eucalyptol (1,8-cineole), would be represented by the largest peak in the chromatogram, followed by smaller peaks for β-pinene, α-pinene, and camphor, which accounted for 77.23, 6.93, 4.95, and 3.79%, respectively. Oxygenated monoterpenoids (84.05%) and monoterpene hydrocarbons (15.95%) were the main phytochemical classes of *L. coronopifolia* EO.

**Table 1:** Phytochemical compositions of *Lavandula coronopifolia* essential oil, retention time retention index, and formulas.

Name	RT	RI	%	Molecular formula
α-Pinene	8.69	933	**4.95**	C_10_H_16_
Camphene	9.356	949	0.65	C_10_H_16_
Sabinene	10.326	972	1.52	C_10_H_16_
β-Pinene	10.501	476	**6.93**	C_10_H_16_
Myrcene	11.07	990	1.42	C_10_H_16_
Eucalyptol	12.812	1034	**77.23**	C_10_H_18_O
γ-Terpinene	13.912	1060	0.48	C_10_H_16_
Fenchone	15.09	1088	0.22	C_10_H_16_O
α-Thujone	15.6	1101	0.07	C_10_H_18_O
α-Campholenal	16.63	859	0.04	C_10_H_16_O
Trans-Pinocarveol	17.26	1144	0.54	C_10_H_16_O
L-Camphor	17.414	1148	3.79	C_10_H_16_O
Pinocarvone	18.02	1164	0.13	C_10_H_14_O
broneol	18.4	1173	1.03	C_10_H_18_O
Dihydro carveol	19.28	1197	0.69	C_10_H_18_O
Carvone	21	1246	0.31	C_10_H_14_O
Total			100	
Phytochemical groups				
Monoterpene hydrocarbon			15.95	
Oxygenated monoterpenoids			84.05	
Total			100	

Significant values are in bold

The GC–MS chromatogram could be used to identify the individual components of the essential oil, confirm their presence, and determine their relative concentrations. The most abundant components of the oil were eucalyptol (1,8-cineole), β-pinene, α-pinene, and camphor,

### Cytotoxicity effects

As demonstrated by the MTS assay results, *L. coronopifolia* EO has cytotoxic effects against breast cancer (MCF-7), hepatocellular carcinoma (Hep3B & HepG2), and cervical cancer (HeLa) tumor cells in a dose-dependent manner. The cell inhibition percentage is presented in Figure S1 besides the IC_50_ values, which shows the extent to which a substance or treatment reduces the growth or viability of cells, expressed as a percentage of the control cells not treated with the substance or treatment. However, *L. coronopifolia* EO showed the best cytotoxic effect against HepG2 cells with an IC_50_ value equal to 58.51 ± 2.23 µg/mL, as HepG2 cells were strongly affected by several compounds such as *Jugl anthraquinone C* derived from *Juglans mandshurica*, *Anethum graveolens* EO, and other different natural ones^32–34^. This EO was 2-folds more selective to the HepG2 cancer cell line in comparison with the HEK293T cell line, while the selectivity ratio was reduced on the other cell lines (Hep3B, HeLa, and MCF-7) because the IC_50_ values were 489.22 ± 1.89, 444.77 ± 2.4 and > 500 µg/mL for Hep3B, HeLa, and MCF-7 cancer cell lines respectively. Our objective was achieved effectively using observing the apparent inhibitory impact while avoiding the induction of cellular demise or the attainment of saturation. The observed differences in IC_50_ values across various cancer cell lines and HEK293T cells offer significant insights into the inhibitory characteristics of *Lavandula Coronopifolia* Essential Oil. This data holds significance as it aids in the identification of particular cancer subtypes that may be more susceptible to targeted treatment with the essential oil. The evaluation of IC_50_ values in diverse cancer cell lines and normal cells contributes to comprehending essential oils' relative potency and selectivity based on their composition. The observations mentioned above have prospective ramifications for precision cancer treatments and the biophysical characteristics of AMPA receptors, opening avenues for additional research endeavors.

### *Lavandula coronopifolia* did not possess an inhibition effect on AMPAR subunits' cell currents

**Figure 2:**
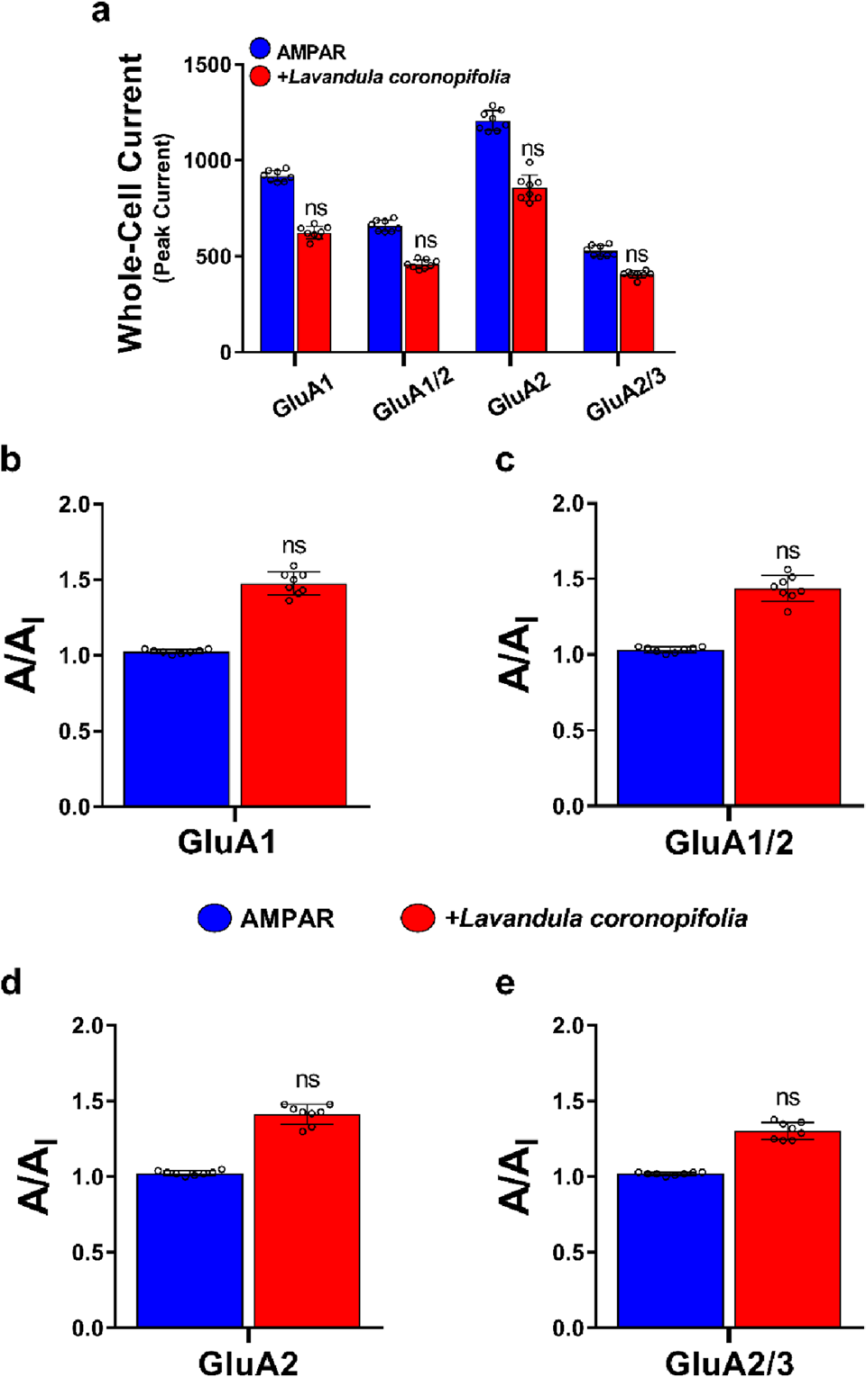
The effect of *Lavandula coronopifolia* essential oil on AMPA receptor homomeric and heteromeric subunits' currents. Figure (**a**) shows the whole-cell recordings of amplitudes (pA) obtained from HEK293T-expressing AMPAR-type subunits (i.e., GluA1, GluA1/A2, GluA2, and GluA2/A3), conducted at − 60 mV, pH 7.4, and 22 °C after treating the cell with 10 mM glutamate (blue) alone, and Glu with a fixed concentration of 120 μM of *Lavandula coronopifolia* oil (red) for 500 ms. *Lavandula coronopifolia* oil concentration was chosen for its highest effect without affecting the health of the cells. Figures (**b, c, d,**) and **e** show the A/A_I_ ratio, where A represents the current generated by glutamate alone, and A_I_ represents the current caused by glutamate + *Lavandula coronopifolia* oil. Data shown are mean ± SEM; n = 8 (number of patch cells in the whole-cell configuration). Significance was calculated using one-way ANOVA, ns, not significant.

The whole-cell patch-clamp technique was used to investigate transfected cells' current changes to evaluate if L. coronopifolia EO inhibits homomeric and heteromeric AMPAR-subunits (i.e., GluA1 and GluA1/A2, GluA2 and GluA2/A3). Glutamate (10 mM) was first administered to the cell for 500 ms to collect evoked current measurements, and then the cells were exposed to the *L. coronopifolia* EO solution. Before exposure to the *L. coronopifolia* EO, the current values were represented by *A*, whereas those following exposures to the oil were represented by *A*_*I*_ (all the data analyses are shown in Table S1). Across all the investigated AMPA-type subunits, there was a slight reduction in current in amplitudes (*A*_*I*_) (Table S1); however, this decrease in currents in all tested AMPAR subunits was insufficient to be considered AMPA receptor inhibition since the A/A_I_ ratio was less than twofold. The *L. coronopifolia* EO dropped all homomeric and heteromeric subunits' amplitude almost onefold (Fig. 2).

### The *Lavandula coronopifolia* essential oil affects AMPAR biophysical gating properties

**Figure 3:**
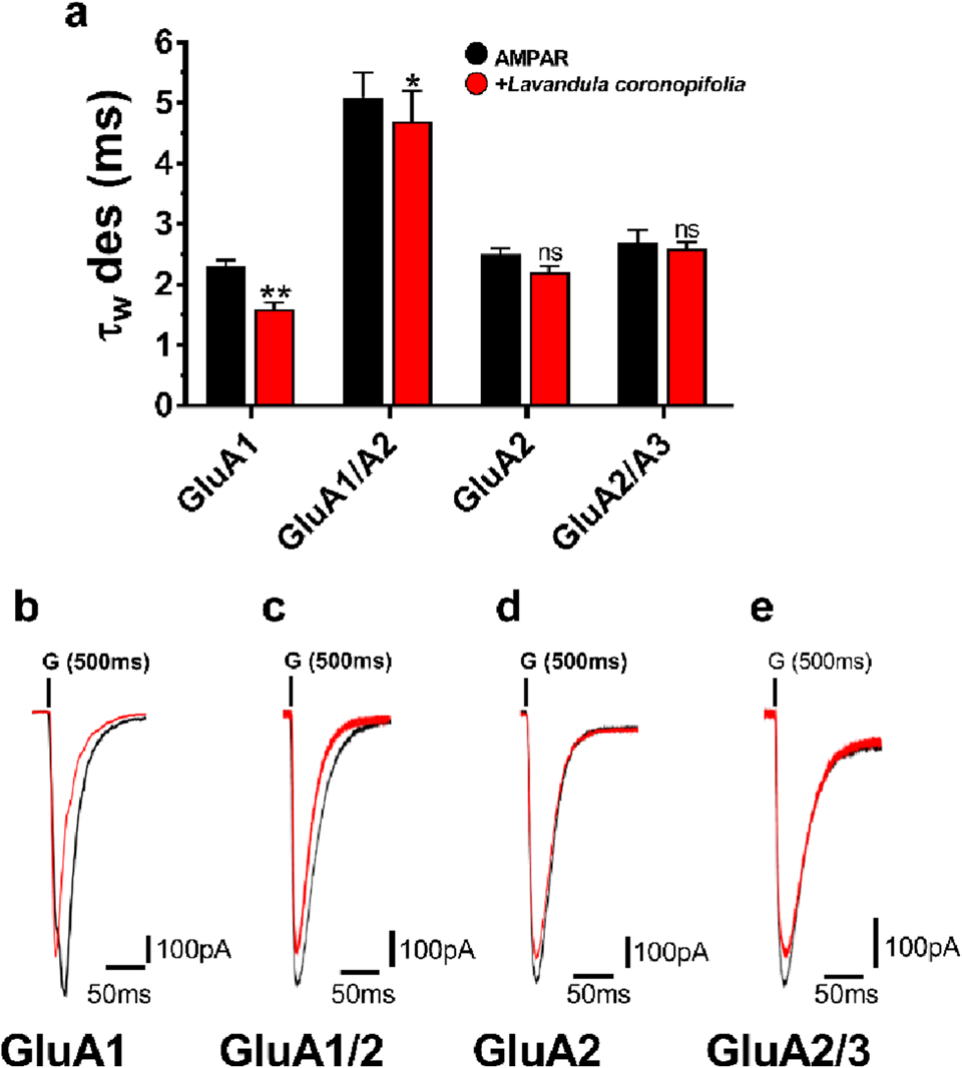
*Lavandula coronopifolia* effect on AMPAR subunits desensitization rate. *Lavandula coronopifolia* oil modifies AMPARs desensitization time (τ_w_ des) from HEK293T cells expressing AMPAR-type subunits (GluA1, GluA1/A2, GluA2, and GluA2/A3) upon 500 ms. Figure (**a**) show the whole-cell current recording that was conducted by exposing AMPAR-type subunits to glutamate (Glu) alone (10 mM) (black) or Glu with *Lavandula coronopifolia* oil at a fixed concentration of 120 μM (red). Figures (**b, c, d**,) and **e** show the traces obtained in the presence (red) and absence (black) of *Lavandula coronopifolia* oil. G represents the 10 mM glutamate used in the experiment, noted above the current trace. Data are shown as mean ± SEM; n = 8 (number of patch cells in the whole-cell configuration). Significance (one-way ANOVA): * *p* < 0.05; ** *p* < 0.01; ns, not significant.

The next step was to ascertain the efficacy of *L. coronopifolia* EO on AMPARs biophysical gating properties to determine pharmacological potencies. One potential therapeutic strategy is the desensitization and deactivation of AMPARs to alleviate persistent excitatory AMPAR activity (Table S1). AMPARs become desensitized when HEK293T cells are exposed to glutamate for 500 ms. Our findings indicate that *L. coronopifolia* EO has affected the tested subunits since post-*L. coronopifolia* EO lowered tau (τ_w_ des) values by about one-fold (Fig. 3). When HEK293T cells were treated with *L. coronopifolia* EO, the desensitization rate of GluA1 decreased by a factor greater than one. Furthermore, the *L. coronopifolia* EO impact is not coupled in homomeric or heteromeric subunits since they are affected almost the same way (Fig. 3).

**Figure 4:**
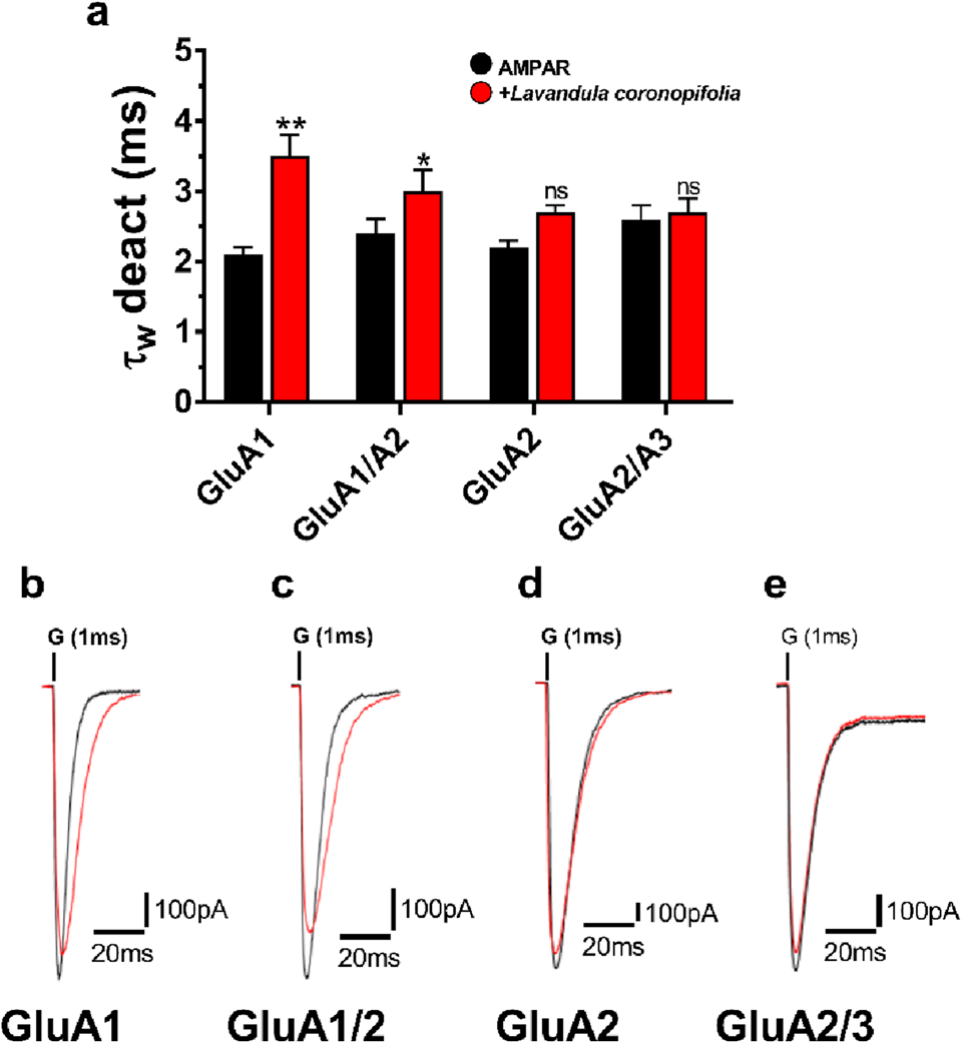
*Lavandula coronopifolia* effect on AMPAR subunits deactivation rate. *Lavandula coronopifolia* oil modifies AMPARs deactivation time (τ_w_ deact) from HEK293T cells expressing AMPAR-type subunits (GluA1, GluA1/A2, GluA2, and GluA2/A3) upon 1 ms. Figure (**a**) show the whole-cell current recording that was conducted by exposing AMPAR-type subunits to glutamate (Glu) alone (10 mM) (black) or Glu with *Lavandula coronopifolia* oil at a fixed concentration of 120 μM (red). Figures (**b, c, d**,) and **e** show the traces obtained in the presence (red) and absence (black) of *Lavandula coronopifolia* oil. G represents the 10 mM glutamate used in the experiment, noted above the current trace. Data are shown as mean ± SEM; n = 8 (number of patch cells in the whole-cell configuration). Significance (one-way ANOVA): * *p* < 0.05; ** *p* < 0.01; ns, not significant.

The deactivation value (τ_w_ deact) for the GluA1 receptor, on the other hand, increased approximately by twofold following the application of the *L. coronopifolia* EO, whereas GluA1/A2, GluA2, and GluA2/A3 values increased around onefold (Fig. 4). The EO of *L. coronopifolia* was generally effective in influencing desensitization and deactivation processes.

## Discussion

This research analyzed the chemical composition of *Lavandula coronopifolia* essential oil, as well as its cytotoxic effects on several cancer cell lines and impact on AMPA receptor subunit cell currents. The pharmacological activities of *Lavandula coronopifolia* EO's chemical components that have been investigated in this research, eucalyptol, β-pinene, α-pinene, and camphor, are well-known for their antibacterial^35^, antifungal^36^, and anti-inflammatory properties^37^. These components, along with the oxygenated monoterpenoids and monoterpene hydrocarbons commonly found in lavender essential oils, have been found to possess various other pharmacological activities, such as antioxidants^38^, antimicrobial, and anticancer effects. In particular, recent studies have shown that *Lavandula coronopifolia* EO has potent cytotoxic effects against various cancer cell lines, including MCF-7, HeLa, HepG2, and Hep3B. Notably, the IC_50_ value of 58.51 ± 2.23 µg/mL for HepG2 cells was lower than that reported for other natural compounds with known anticancer activity, such as Jugl anthraquinone C derived from *Juglans mandshurica* and *Anethum graveolens* EO^32^. Moreover, the selectivity ratio was found to be higher for HepG2 cells compared to other cancer cell lines, suggesting that *Lavandula coronopifolia* EO may hold promise as a selective anticancer agent.

Interestingly, the chemical composition of *Lavandula coronopifolia* EO can vary depending on a range of factors, such as geographical origin, temperature, relative humidity conditions, soil, genetics, and degree of maturity^39^. For instance, a previous study by Aburjai et al. found that 1,8-cineole comprised 7.32–25.43% percent of *L. coronopifolia* EO from Jordan, while oxygenated monoterpenes made up 80.60–85.56%, followed by monoterpene hydrocarbons (5.99–8.12%)^40^. In contrast to our findings, Messaoud et al. reported that trans-ocimene (26.9%), carvacrol (18.5%), bisabolene (13.1%), and myrcene (7.5%) were the major components of the EO of *L. coronopifolia* aerial plant parts (leaves and flowers) from Tunisia and that monoterpene hydrocarbons (46.2%) were the most abundant group, followed by oxygenated monoterpenes^38^. Similarly, Hassan et al. studied the *L. coronopifolia* EO from Saudi Arabia and identified phenol-2-amino-4,6-bis (1,1-dimethyl ethyl) (51.18%) as the main constituent. These findings highlight the variability of *Lavandula coronopifolia* EO's chemical composition and underscore the importance of considering these factors when assessing its pharmacological properties^8^.

Overall, the combination of potent pharmacological effects and varied chemical composition of *Lavandula coronopifolia* EO makes it an intriguing target for future research and therapeutic use. A future study might concentrate on discovering the precise chemical components responsible for its anticancer potential, as well as finding the best cultivation and extraction conditions to maximize yield and potency.

AMPARs are abundant on dendritic spines' postsynaptic membranes, are highly active, and shuttle in and out of synapses. When glutamate attaches, AMPARs are activated, opening the pore and enabling the entry of Na^+^ ions (together with K^+^ efflux) to depolarize the postsynaptic compartment^41,42^. Ca^2+^ influx is also facilitated by AMPARs, which has significant consequences for plasticity via activating Ca^2+^-dependent signaling systems^43^. AMPAR plasticity dysregulation has been linked to various clinical conditions, including Alzheimer's disease, autism spectrum disorders, Parkinson's disease, epilepsy, amyotrophic lateral sclerosis (ALS), ischemia, and drug addiction^44^. AMPARs overactivation is potently excitotoxic, causing immediate or delayed neurotoxicity, and has significant implications for mental health^45^.

This study tested the *L. coronopifolia* EO on homomeric and heteromeric subunits. Heterotetramers of AMPARs are the most common form of brain receptors, considerably increasing the number of functional subtypes. Homomers of AMPARs are possible, although favored heteromers of GluA1–GluA4 in different combinations are preferred^46^. The GluA2 subunit is found in many AMPA receptor complexes, limiting Ca^2+^ permeability and low single-channel conductance values^47^. Besides, GluA1-containing AMPA receptors will undoubtedly shed light on novel therapeutic strategies for treating dementia and age-related cognitive disorders of Alzheimer's.

It was essential to investigate and comprehend the biophysical gating properties of AMPA receptors to treat the diseases stated. AMPAR measures are empirical measures of current decay from the active state to the deactivation of AMPARs. The measured decay of current following glutamate removal after a short stimulation time determines deactivation^48^. At the same time, desensitization is a kinetic property of receptors in which channels become liganded but closed (desensitized) over a predictable time. Auxiliary subunits and RNA splicing control the degree of desensitization, which has essential physiological roles of glutamate depending on the kinetics of glutamate in the synaptic cleft and is involved in protecting neurons from the neurotoxic effect^49^. Calcium influx via AMPARs is uncontrolled when AMPAR subunit composition, function, or desensitization kinetics are impaired; downstream pathways are overactivated^50^. Subsequently, managing deactivation and desensitization, essential in creating the synaptic response, could contribute to the development of cancer treatments.

## Conclusion

Significant amounts of eucalyptol, β-pinene, and α-pinene were discovered in the EO extracted from the leaves of *L. coronopifolia*. In vitro, cytotoxicity studies revealed that the EO was potentially cytotoxic, notably in HepG2 cancer cells, and inhibited these cancer cell lines twice as selectively as HEK293T cell lines. *L. coronopifolia* EO also displayed neuroprotective effects, showing its future potential as a reliable source of phytopharmaceuticals. Preclinical studies on *L. coronopifolia* EO would be required to establish its safety and effectiveness, but the findings reported here are intriguing and suggest promising possible future applications.

## Supplementary Information

Supplementary Information.

## Data Availability

The article includes all the utilized data to support the current study's findings.

## Abbreviations

EOs: Essential oils
*L. coronopifolia*: *Lavandula coronopifolia*
*AD*: Alzheimer's disease
AMPA: α-Amino-3-hydroxymethyl-4-isoxazolyl-propionic acid
AMPAR: α-Amino-3-hydroxymethyl-4-isoxazolyl-propionic acid receptor
HEK293T: Human embryonic kidney
ANOVA: One-way analysis of variance
Glu: Glutamate
ALS: Amyotrophic lateral sclerosis
