# Supplementary material for: The effect of Lavandula Coronopifolia essential oil on the biophysical properties of desensitization and deactivation gating currents in ionotropic receptors
Source: Sci Rep. 2023 May 24;13:8417. doi: 10.1038/s41598-023-35698-0 (PMC10209083; doi:10.1038/s41598-023-35698-0)
Supplement: Supplementary file 1 — Supplementary Information. [file 41598_2023_35698_MOESM1_ESM.docx]

**Supplementary material**

**The Effect of *Lavandula Coronopifolia* Essential Oil on the Biophysical Properties of Desensitization and Deactivation Gating Currents in ionotropic receptors**

**Mohammad Qneibi^1,^*, Nidal Jaradat^2,*^, Nawaf Al-Maharik^3^, Mohammed Hawash^2^, Linda Issa^2^, Shorooq Suboh^1^, Leen Yahya^2^, Adan Abu Khait^2^, Amjaad Warasneh^2^, Sosana Bdir^1^**

^1^ Department of Biomedical Sciences, Faculty of Medicine and Health Sciences, An-Najah National University, Nablus, Palestine.

^2^ Department of Pharmacy, Faculty of Medicine and Health Sciences, An-Najah National University, Nablus, Palestine.

^3^ Department of Chemistry, Faculty of Sciences, An-Najah National University, Nablus, Palestine.

Corresponding Authors:

Corresponding Author: Mohammad Qneibi, Tel. +00-972-545 975 16; Fax. +00-970-9 2345 982; E-mail address: mqneibi@najah.edu; Postal address: P.O.Box 7 Nablus-Palestine

# Table of Contents

**Table S1. Whole-Cell Recordings………………………………………..…………………..3**

**Figure S1. Cytotoxic effects of the EO against MCF-7, HeLa, HepG2, and Hep3B cancer cell line compared to HEK293T cell lines……………………………………………….….4**

**Table S1. Whole-Cell Recordings**

Data shown are mean ± SEM; n = 8 (number of patch cells in the whole-cell configuration). A one-way ANOVA test was used to measure the significance and set as: * p < 0.05; ** p < 0.01; ns, not significant.

| **Receptor Name/Compounds abbreviation** | **GluA1 (Glutamate Alone)** | **Lavandula coronopifolia** | **Applying Glutamate Alone After Lavandula coronopifolia** | **n** | **A/A_I_** |
| --- | --- | --- | --- | --- | --- |
| **Amplitude** | 920±38 | 633±42^ns^ | 899±39 | 8 | 1.48±0.1 |
| **(pA)** |  |  |  |  |  |
| **t deact** | 2.1±0.1 | 3.5±0.3^**^ | N/R | 8 | N/R |
| **(ms)** |  |  |  |  |  |
| **t des** | 2.3±0.1 | 1.6±0.1^**^ | N/R | 8 | N/R |
| **(ms)** |  |  |  |  |  |
| **Receptor Name/Compounds abbreviation** | **GluA1/2 (Glutamate Alone)** | **Lavandula coronopifolia** | **Applying Glutamate Alone After Lavandula coronopifolia** | **n** | **A/A_I_** |
| **Amplitude** | 660±37 | 501±29^ns^ | 640±36 | 8 | 1.44±0.1 |
| **(pA)** |  |  |  |  |  |
| **t deact** | 2.4±0.2 | 3.0±0.3^*^ | N/R | 8 | N/R |
| **(ms)** |  |  |  |  |  |
| **t des** | 5.1±0.4 | 4.7±0.5^*^ | N/R | 8 | N/R |
| **(ms)** |  |  |  |  |  |
| **Receptor Name/Compounds abbreviation** | **GluA2 (Glutamate Alone)** | **Lavandula coronopifolia** | **Applying Glutamate Alone After Lavandula coronopifolia** | **n** | **A/A_I_** |
| **Amplitude** | 1209±68 | 933±46^ns^ | 1179±72 | 8 | 1.41±0.1 |
| **(pA)** |  |  |  |  |  |
| **t deact** | 2.2±0.1 | 2.7±0.1^ns^ | N/R | 8 | N/R |
| **(ms)** |  |  |  |  |  |
| **t des** | 2.5±0.1 | 2.2±0.1^ns^ | N/R | 8 | N/R |
| **(ms)** |  |  |  |  |  |
| **Receptor Name/Compounds abbreviation** | **GluA2/3 (Glutamate Alone)** | **Lavandula coronopifolia** | **Applying Glutamate Alone After Lavandula coronopifolia** | **n** | **A/A_I_** |
| **Amplitude** | 530±33 | 399±26^ns^ | 519±34 | 8 | 1.30±0.1 |
| **(pA)** |  |  |  |  |  |
| **t deact** | 2.6±0.2 | 2.7±0.2^ns^ | N/R | 8 | N/R |
| **(ms)** |  |  |  |  |  |
| **t des** | 2.7±0.2 | 2.6±0.1^ns^ | N/R | 8 | N/R |
| **(ms)** |  |  |  |  |  |

**Figure S1.** Cytotoxic effects of the EO against MCF-7, HeLa, HepG2, and Hep3B cancer cell line compared to HEK293T cell lines.
